# Grand Challenges in Gene and Epigenetic Editing for Neurologic Disease

**DOI:** 10.3389/fgeed.2019.00001

**Published:** 2020-01-23

**Authors:** David J. Segal

**Affiliations:** Department of Biochemistry and Molecular Medicine, Genome Center, University of California, Davis, Davis, CA, United States

**Keywords:** gene editing, epigenetic editing, neurologic disease, brain, neurons, genetic medicine

The term genetics may evoke images of nucleic acid sequences for most researchers. However, the pragmatic filed of medicine might consider it as information about a patient that can be categorized as actionable, deterministic, probabilistic, or a variant of uncertain significance. Actionable information is any information that can inform a clinical decision, such as *BRCA1/2* status informing a decision for prophylactic surgery. However, actionable information is currently the smallest category. There are over 6,000 single-gene disorders, the vast majority of which have no targeted clinical treatment. Such variants are merely deterministic, increasingly discoverable even before birth by a cell-free fetal DNA test but unable to inform any treatment that could alter the disease trajectory. A good example is the APOE ε4 allele, which imparts a high risk of Alzheimer's disease, but currently informs no action that can change clinical outcome. Today, the discussion is whether it is ethical to test for such variants that could provide only a burden with little hope for benefit. An even greater numbers of less-penetrant variants are either certain to increase the probability of disease or have uncertain significance. Certainly, a grand challenge for all of genetic medicine over the next decade will be to move more of this information into the actionable category.

## Challenges for Delivery Vectors

Fortunately, the potential to advance toward this goal has never seemed more achievable, even for the historically challenging field of neurologic disease. Indeed, the very first gene therapy approved in the United States was designed to treat inherited retinal dystrophy by transfer of wild-type *RPE65* to the retinal pigmented epithelium of the eye (High and Roncarolo, 2019). Approved by the FDA in December 2017, Luxturna ushered in the long-anticipated era of gene therapy for diseases of the central nervous system (CNS). Adeno-associated virus (AAV), such as the AAV2 used in Luxturna, has become the vector of choice for delivery of many *in-vivo* gene therapy and gene editing applications, although other capsid proteins such as the serotype 9 (recombinant AAV2/9 or simply AAV9) are generally more efficacious for neuronal transduction in organs such as the brain (Ingusci et al., 2019). However, efficient delivery of genes and gene editing tools into neurologic tissues remains perhaps *the* most significant challenge for treatments of neurological diseases.

### Delivery by Viral Vectors

The challenge of delivery can be subdivided into interrelated subcategories such as immunogenicity, biodistribution, and transduction efficiency. Importantly, there are no objectively optimal parameters for these factors; rather, the parameters will be dependent on the specific application. For example, immunogenicity may be highly dependent on the route of administration. Unlike mice, humans readily produce antibodies to AAV which, if not precluding the use of an AAV because of preexisting antibodies, will likely prevent re-administration of the same AAV serotype. Injection of the AAV directly into the brain parenchyma or pockets of cerebrospinal fluid (CSF) are one strategy to limit immune response (e.g., Gray et al., 2013). However, it has been difficult to obtain brain-wide distribution with such direct injections in most studies (Hinderer et al., 2018; Ohno et al., 2019). One of the most significant recent advances in this field was the *in-vivo* evolution of a new AAV capsid protein variants that could efficiently cross the blood-brain barrier, including PHP.B (Deverman et al., 2016) and subvariant PHP.eB (Chan et al., 2017). This discovery enabled a systemic route of administration, which could provide excellent brain-wide distribution due to entry across the extensive vasculature of the brain. However, systemic administration historically results in high transduction of AAV in the liver, often producing limiting toxicity that must be attenuated by a liver de-target strategy. In addition, systemic delivery again exposes the AAV to immune responses in the periphery. Thus, the development of efficient brain-wide, non-toxic, non-immunogenic delivery systems for genes and editing tools remains a major unmet challenge, particularly in primates and humans for which transduction by PHP.B and PHP.eB are currently far less efficient.

### Delivery by Non-viral Vectors

A promising alternative to viral vectors is synthetic nanoparticle delivery systems, such as the lipid nanoparticles used for hepatic delivery of the first FDA approved small interfering RNA (siRNA) drug Onpattro (Patisiran) (Adams et al., 2018). It has been more challenging to achieve efficiency delivery of larger cargos such as *Sp*Cas9 as protein (molecular weight 158,441 g/mole) or mRNA (1,333,800 g/mole), though this is starting to change (Jiang et al., 2017; Miller et al., 2017; Finn et al., 2018). However, lipo-particle delivery has been largely restricted to the liver.

Nonetheless, the conceptually infinite potential to engineer these particles provides great hope that such systems could 1 day supplant viral vectors for therapeutic applications. In principle, the outer surfaces can be decorated with binding ligands to target them to specific cell types. Other types of non-viral systems are also in development. Purified zinc finger-based artificial transcription factors have been reported to activate genes in the brain upon systemic injection in mice (Bailus et al., 2016). Similar gene editing proteins could be produced by cells in the body, such as transplanted stem cells or virally transduced endogenous cells, and introduced into neighboring cells by secretion and reuptake or transport by extracellular vesicles.

## Challenges of Targeting Specific Cells

While brain-wide transgene integration or editing may be useful for many therapeutic applications, other uses face the challenge of delivery to specific cell types, such as studying the role of particular neurons in a disorder or manipulating specific neural circuits. In principle, there are three conceptual mechanisms by which particular cells can be specified: *outside, inside*, and *location*. Viral “tropism” is largely dependent on the presence of particular cell-surface receptors on *outside* of the target cells. Many efforts have focused on substituting (e.g., pseudotyping lentiviral vectors with the pantropic VSV-G envelope protein; Okimoto et al., 2001), augmenting (e.g., using bi-specific antibodies), or evolving (e.g., the Cre-dependent selection methods used to obtain PHP.B; Deverman et al., 2016) interactions with cell-surface proteins. These approaches hold great promise for future advances in the recognition of specific cell types, but are limited by the comparatively few proteins expressed on the surface compared to all cellular proteins and the fact that these proteins are often not unique to specific cell types. A richer source of specificity information is the unique transcriptome and proteome *inside* the cell, which is essentially the biochemical basis differentiating one cell type from another. Historically this *inside* information has been exploited by using tissue-specific promoters and enhancers (Fitzsimons et al., 2002) or activity-dependent promoters (Kawashima et al., 2013). However, true cell-type level specificity has been difficult to achieve. Future work is envisioned to extend such specificity mechanisms to include non-coding RNAs, such as micro-RNAs (Wang et al., 2019) and non-transcription-factor endogenous proteins, as well as combinations of such *inside* molecules with *outside* information. However, a potential third source of specificity information that as yet has been only superficially explored is *location*. Apart from blood and some other migratory cells, the vast majority of cells have very precise locations within tissues. Traditionally, neurological research has taken the lead in location-based target specificity due to extracerebral stereotactic landmarks such as bregma, although these methods have thus far been limited to specifying regions of the brain such as the hippocampus or hypothalamus. However, other methods for using physical location are in their infancy, such as the use of nanomagnets (Zhu et al., 2019) or focused ultrasound (Fisher and Price, 2019) to guide or release editing tools from nanoparticles at focal locations. Additional innovative and technologically sophisticated methods could be developed in the future. Significant advances in these areas are needed to move us beyond tissue-specific promoters to true cell-type-specific and potentially cell-specific applications.

## Challenges of Gene and Epigenetic Editing in the Brain

### Nuclease-Mediated Gene Editing in the Brain

By now, the paradigm of nuclease-mediated gene editing has been well-established. Engineered meganucleases, zinc finger nucleases (ZFNs), TAL effector nucleases (TALENs), CRISPR/Cas9 nucleases from various species and the related CRISPR/Cas12 (formerly Cpf1) from various species can all be programmed to create double-strand breaks (*DSBs)* at precise sequences in human cells such as neurons (Maeder and Gersbach, 2016; Cota-Coronado et al., 2019). Non-Homologous End Joining (NHEJ) is the predominant repair pathway for such DSBs, which we now know are usually repaired perfectly (Nelson et al., 2016). However, perfect repair recreates the target, enabling additional rounds of cleavage until low-frequency short insertion or deletions (indels) occur that disrupt the target site. Using this approach to create NHEJ indel mutations in exons of coding genes to knock-out gene expression has been the major use of nucleases *in vivo*. The potential for off-target events in gene editing was described early on, and while there are still no standardized method to identify off-targets, a number of strategies to identify and minimize off-target continue to emerge (Zischewski et al., 2017; Kimberland et al., 2018; Wienert et al., 2019). Addition of a donor DNA with homology to the target site can provide information on how to repair the DSB can allow the correction or insertion of DNA sequence by homology-dependent repair (HDR). However, the enzymes required for HDR are only expressed in the S, G2, and early M phases of the cell cycle, precluding the use of HDR for non-dividing cells such as mature neurons. Realization that the DSB is usually repaired perfectly allowed for the development of efficient and precise targeted insertions through methods such as homology-independent targeted insertion (HITI), homology-mediated end joining (HMEJ), and others (Liang et al., 2008; Yao et al., 2017, 2018; Suzuki and Belmonte, 2018). Other factors important for gene editing in the brain concern the method and physical form of nuclease delivery. Viral vectors can provide efficient delivery, but long-term expression of nucleases dramatically increase off-target cleavage events. For that reason, mRNA or purified ribonucleoprotein (RNP) are the preferred delivery forms, or a mechanisms to inactivate viral expression. However, more recent studies have revealed that there are unexpected on-target events that may be even more concerning, highlighting the basic tenet that in science we only see what we look for. Such events include death of some primary cells that are sensitive to double-strand breaks, non-targeted large deletions of up to several kilobases, and genomic rearrangements including the loss of entire chromosome arms (Haapaniemi et al., 2018; Ihry et al., 2018; Kosicki et al., 2018; Wang et al., 2018; Cullot et al., 2019; Nelson et al., 2019). Therefore, the efficient correction of mutations in the billions of neurons in the CNS remains a major challenge.

### Beyond DSBs: Base Editing, Prime Editing, Transposon Editing

DSB repair pathways have guided the technology of gene editing since the earliest reports of gene targeting in the 1980s (Thomas and Capecchi, 1986). However, advances from the lab of David Liu at Harvard and others have provided efficient tools to usurp other types of DNA repair. Cytidine or adenosine deaminases convert C to U and A to hypoxanthine in single-stranded nucleic acids, respectively. Tethering these enzymes to zinc finger and TALE proteins had been envisioned earlier, but only became efficient with the advent of Cas9 due to the single-stranded DNA that is displaced by guide-RNA binding. An additional advance was to use a nickase version of the Cas9 to stimulate the DNA mismatch repair pathway to favor the desired repair, enabling highly efficient *base editing* of C:G base pairs to T:A, and A:T to G:C (Komor et al., 2016; Gaudelli et al., 2017). These pathways are active in non-dividing cells, allowing potential correction of as much as 60% of known human pathogenic single-base mutations without DSBs or donor DNAs. An even more recent Cas9 nickase application is *prime editing*, which again nicks the displaced DNA but uses its 3′ end as a primer for reverse transcription of short sequences brought in on the tail of the guide-RNA (Anzalone et al., 2019). Though this approach is in its infancy, it could similarly enable highly efficient prime editing of any substitution of one or a few bases, or small insertions/deletions (indels). An alternative strategy to nickase approaches is for the tool to perform more of the double-strand break and resealing process. This has also been shown recently with report of Cas9-based transposases, which are able to integrate larger sequences of DNA into target sites and potentially mediate other rearrangements (Klompe et al., 2019; Strecker et al., 2019). However, larger protein complexes and donors may face more complex delivery challenges. It is unknown at the time of this writing if nuclease-based gene editing has reached an inflection point and these new methods will take over for gene manipulations in the CNS. We should expect these tools will be thoroughly tested over the next few years, and anticipate similarly innovative new additions to the toolbox.

### No Breaks: Gene Regulators, Epigenetic Editing, and RNA Editing in the Brain

On the timescale of individual organisms, nature generally does not regulate gene expression by changing DNA sequence. Rather, it uses a complex interplay of *trans* regulators and *cis* epigenetic information. The same basic design developed for the first targetable artificial transcription factors (Beerli et al., 1998) are still in use today, tethering a KRAB transcriptional repression domain or VP16-like transcriptional activation domain to a programmable DNA binding platform, with several notable variations in the structure and arrangement of effector domains (Thakore et al., 2016). However, the effects of these tools tend to be *transient*, regulating gene expression only while the protein is bound to the DNA. Other old (e.g., siRNA, ASO) and new (e.g., Cas13, a targetable RNA nuclease; Abudayyeh et al., 2017) can transiently inhibit gene expression by targeting its continuously produced mRNA. These transient repressors can be made persistent by expressing them constitutively, such as from a viral vector (e.g., dCas9-KRAB, shRNA, or Cas13). However, as discussed above, long-term expression of a foreign (in these cases, bacterial) transgene can lead to T-cell mediated immune responses against the expressing cells. An alternative to persistence by long-term expression would be persistence by long-term effect. In nature, long-term modifications in gene expression, such as silencing liver enzymes in CNS neurons over the lifetime of an individual, is accomplished by changes in epigenetic information such as histone and DNA methylation. Understanding the enzymes that write (e.g., DNMT3A, EZH2, HAT1) and cause the erasure of (e.g., TET1/2, HDAC1, LSD1) this type of information has enabled the creation of targetable epigenetic editors (Cano-Rodriguez and Rots, 2016; Bashtrykov and Jeltsch, 2017). In some impressive examples, these tools have facilitated the complete repression of gene expression that is *persistent* over multiple cell divisions (Amabile et al., 2016; Saunderson et al., 2017; Wei et al., 2019). However, more generally what was learned is that we do not yet fully understand how to transition from one epigenetic state to another in a predictable and robust manner. Intense efforts are needed to fully realize the potential of persistent epigenetic editing for CNS disorders.

## The Challenge of What to Edit: The Final Frontier in Gene Editing for Neurologic Disease

### Neurologic Disorders of Known Genetic Ideology

The first set of targets will almost certainly be focused on correcting or bypassing mutations that cause genetic diseases. Several gene editing companies have publicly disclosed CNS programs that will likely be the first to enter clinical trials, such as Editas Medicine (editasmedicine.com) for Leber Congenital Amaurosis 10 and Usher Syndrome 2a, and Sangamo Therapeutics (sangamo.com) for tauopathies (e.g., Alzheimer's disease), amyotrophic lateral sclerosis (ALS), and Huntington's disease. Other preclinical gene editing studies have been reported for numerous CNS-related disorders, and there will be more to come. As techniques improve, it would not be unexpected to eventually see studies attempting to rescue, for example, all known mouse models of epilepsy or all known autism spectrum disorder genes. We will see studies using multiple guide-RNAs to target several disease genes simultaneously.

### Beyond Broken Wires

Beyond ameliorating rare genetic disorders, there will likely be many applications about which today we can only speculate. There is a longstanding philosophy among some neuroscientists that neurons might be considered as mere wires in a circuit; the wires may be broken (due to genetic mutations) but if they are intact the only property of interest is their connections and not their genetics. However, there is emerging evidence that differential gene expression within neurons can affect specific behaviors. Neonatal rats weaned under early life stressful conditions display depression-like and anxiety-like behaviors and have significant differentially methylated regions (DMRs) in their brains as adults (Singh-Taylor et al., 2018). Reduced *NTRK3* expression in the dorsal amygdala of rhesus macaques is associated with increased early-life anxious temperament, which could be reversed by increasing NTRK3 signaling using AAV5 delivery of NTRK3's natural ligand (Fox et al., 2019). Such observations raise questions as to the extent that certain behaviors might one day be controlled by targeted genetic or epigenetic regulation in the CNS. Put plainly, can a bad experience be erased using molecular genetics? Certainly such an advance could have tremendous impact for more common psychological conditions such as anxiety (affecting 18% of US population), major depression (6.7%), post-traumatic stress disorder (3.5%), and autism spectrum disorder (1.4%) (ADAA, 2019) What might be the social, legal and ethical implications of such capabilities? Perhaps the greatest challenges lie in the near future, as tools and delivery methods reach an efficiency allowing for long-term alteration of gene expression in subsets of specific neurons. Such capabilities will enable us, for the first time, to precisely probe the relationships between our genes and our complex behaviors.

## Author Contributions

The author confirms being the sole contributor of this work and has approved it for publication.

### Conflict of Interest

The author declares that the research was conducted in the absence of any commercial or financial relationships that could be construed as a potential conflict of interest.
